# Is there an association between hysterectomy and subsequent adiposity?

**DOI:** 10.1016/j.maturitas.2007.09.004

**Published:** 2007-11-20

**Authors:** Rachel Cooper, Diana Kuh, Rebecca Hardy, Chris Power

**Affiliations:** a Centre for Paediatric Epidemiology and Biostatistics, Institute of Child Health, University College London, UK; b MRC National Survey of Health and Development, Department of Epidemiology and Public Health, University College London, UK

**Keywords:** Hysterectomy, Consequences, Body mass index, Waist circumference, Adiposity

## Abstract

**Objectives:**

To examine the associations between hysterectomy and subsequent adiposity and to investigate whether these associations vary by characteristics of hysterectomy and are independent of pre-hysterectomy adiposity and potential confounding factors.

**Methods:**

Using information on women from the 1946 and 1958 British birth cohort studies (*N* = 1790 and 4552, respectively), collected prospectively across life, regression analyses were used to examine the associations between hysterectomy and subsequent body mass index (BMI) and waist circumference.

**Results:**

In unadjusted analyses there was a difference of 1.18 kg/m^2^ (95% CI: 0.64, 1.74) in mean BMI and of 2.72 cm (1.45, 3.99) in waist circumference at age 44–45 years between women who had undergone hysterectomy and those who had not in the 1958 cohort, and differences of 0.76 kg/m^2^ (−0.05, 1.57) and 0.34 cm (−1.58, 2.26) at age 43 years and 0.81 kg/m^2^ (0.14, 1.49) and 1.45 cm (−0.15, 3.05) at age 53 years in the 1946 cohort. These differences attenuated and were no longer significant after adjustment for pre-hysterectomy BMI and confounders. There was no strong evidence of variation in associations by oophorectomy status, timing, route of or reason for procedure.

**Conclusions:**

This study demonstrates that British women who had previously undergone hysterectomy had higher BMI and waist circumference in middle-age than others. These differences appear to be accounted for by the higher BMI in earlier adulthood and increased levels of risk factors associated with both adiposity and hysterectomy risk among women who had undergone hysterectomy. This suggests that women are unlikely to gain weight as a direct result of hysterectomy.

## 1 Introduction

The evidence for an association between hysterectomy and subsequent adiposity is unclear and unconvincing. It has been suggested that hysterectomy could cause weight gain. This is plausible given that hysterectomy could result in hormonal changes or disruption to fat and muscle tissue in the abdomen during surgery, or recuperation after hysterectomy could result in a period of inactivity. However, most studies [1–4] that have attempted to examine whether hysterectomy affects subsequent adiposity have used cross-sectional data. This is inappropriate as it is not possible to establish from these studies in which direction the association is acting—if women who have undergone hysterectomy are found to have higher body weight than other women this could be explained by an effect of hysterectomy on subsequent body weight or by women who undergo hysterectomy having higher body weight than other women even prior to surgery. This latter explanation is also plausible as evidence suggests that adiposity may influence subsequent risk of gynaecological disorders [5,6] and hysterectomy [7,8]. Ideally, to overcome the problem associated with using cross-sectional data and to establish whether there is a relationship between hysterectomy and subsequent adiposity, longitudinal studies with measures of adiposity taken before and after hysterectomies are needed. Existing longitudinal studies [9–13] are limited by a reliance on self-reported outcome measures, short follow-up, small sample size, different methods of data collection and/or a lack of control for confounding factors.

The main objectives of this study were to examine whether hysterectomy is associated with subsequent BMI and waist circumference, to investigate whether associations are explained by pre-hysterectomy BMI and are independent of factors across life that potentially confound the associations and to assess whether there is variation in associations by characteristics of hysterectomy. We use data from the 1946 and 1958 British birth cohort studies that have been collected prospectively across life from birth, enabling us to clarify the temporal relationship between hysterectomy and BMI and waist circumference. Comparing the findings from these two birth cohorts is of benefit as rates of hysterectomy [14] and levels of overweight and obesity in the UK [15] have changed over time whereby it cannot be assumed that associations found in one birth cohort will necessarily apply to others.

## 2 Methods

### 2.1 Study populations

The 1946 birth cohort, also known as the Medical Research Council National Survey of Health and Development, is a socially stratified cohort of 5362 males and females followed up since their births across England, Scotland and Wales during 1 week in March 1946 [16]. Of the 2547 females in the original cohort, 1797 (70.6%) women have participated in at least one data collection since 1989.

The 1958 birth cohort, also known as the National Child Development Study consists of 17,638 males and females followed up since their births across England, Scotland and Wales during 1 week in March 1958. To ensure national representativeness 920 immigrants to the UK born during the same week in March 1958 were recruited into the study up to age 16 years [17]. Of the 8959 females in the cohort, 6002 were included in the target sample for a recent biomedical survey, which took place at age 44–45 years, of whom 4712 participated.

### 2.2 Ascertainment of outcome measures

BMI (kg/m^2^) was calculated using height and weight measurements taken during physical examinations performed by nurses at the study participants’ homes at ages 43 and 53 years in the 1946 cohort and at age 44–45 years in the 1958 cohort (Table 1). Waist circumference was measured during the same home visits. In the 1958 cohort we used self-reported weights (*n* = 56) and heights (*n* = 54) if consent for measurement was not provided or if the nurse judged that there were difficulties in obtaining accurate measurements.

### 2.3 Ascertainment of hysterectomy status

In the 1946 cohort women were asked about their hysterectomy and oophorectomy status at ages 43 and 53 years; annual reports were also obtained between ages 47 and 54 years, and at 57 years. Women in the 1958 cohort provided similar information during the biomedical survey at age 44–45 years (Table 1).

Hysterectomy was defined as any self-report with or without oophorectomy and in the majority of analyses was grouped into two categories: no hysterectomy or oophorectomy before outcome measurement; hysterectomy with or without oophorectomy before outcome measurement. We excluded women with unknown dates of procedure (*n* = 5, 1946 cohort and *n* = 7, 1958 cohort) and those who had undergone an oophorectomy only (*n* = 30, 1946 cohort and *n* = 67, 1958 cohort), except when examining outcome by oophorectomy status. Timings of operations were reported from which we calculated age at hysterectomy. In the 1946 cohort, information on reason for and route of operation was also available. Reason for hysterectomy was ascertained from hospital records or where these were not available (*n* = 150) from responses to a self-completion questionnaire sent in 2005. Route of hysterectomy was ascertained from women's responses to questionnaires sent between 1997 and 2000 and in 2005.

### 2.4 Covariates

‘Pre-hysterectomy’ measures of BMI were identified: at ages 26 and 36 years in the 1946 cohort and ages 23 and 33 years in the 1958 cohort (Table 1).

Factors potentially associated with both hysterectomy risk and lifetime adiposity were identified *a priori* as potential confounders of the association between hysterectomy and subsequent adiposity. These factors and their methods of collection are described in Tables 1 and 2.

### 2.5 Analysis

Using linear regression models we compared differences in mean BMI and waist circumference between women who had undergone hysterectomy with or without oophorectomy by the time of the specified outcome measurement and those who had not (at ages 43 and 53 years in the 1946 cohort and at age 44–45 years in the 1958 cohort). These differences in means are a measure of the association between hysterectomy and subsequent adiposity.

We then compared differences in mean BMI and waist circumference between women who had undergone hysterectomy with or without oophorectomy, categorised by characteristics of hysterectomy, and the reference group of women who had not. Characteristics of hysterectomy considered were oophorectomy status and age at hysterectomy in both cohorts and also route of and reason for procedure in the 1946 cohort. Likelihood ratio tests were used to ascertain whether there was a significant improvement to model fit arising from categorising hysterectomised women by characteristic of hysterectomy.

Using multiple regression the associations between hysterectomy status and subsequent BMI and waist circumference were adjusted in separate models for each of the potential confounding factors (pre-hysterectomy BMI, age at menarche, parity, lifetime socioeconomic position (own and father's occupational class and educational level), exercise and HRT use). We performed likelihood ratio tests to assess interactions between prior BMI and hysterectomy and between HRT use and hysterectomy. ‘Pre-hysterectomy’ BMI measures were included as continuous variables with quadratic terms included where there was evidence of deviation from linearity, all other covariates were included as categorical variables (categorisations used shown in Table 2). For women with complete data on all variables, models were then run in which all covariates (taking BMI at age 26 in the 1946 cohort and at age 23 years in the 1958 cohort as the measures of ‘pre-hysterectomy’ BMI) were entered into the same model, one at a time, until they had all been added.

Relevant ethical approval was received for this study.

## 3 Results

In the 1946 cohort, 1790 women had reported hysterectomy status, and if they had reported surgery provided a valid date. Of these women, 393 (22%) had undergone hysterectomy by age 54 years. In the 1958 cohort, of 4712 women who participated in the biomedical survey in 2003, 4552 reported hysterectomy status, and if they had reported surgery provided a valid date. Of these women, 437 (9.6%) had undergone hysterectomy by age 44 years. While women in the 1958 cohort experienced a slightly higher prevalence of hysterectomy to age 35 years, by age 44 years prevalence of hysterectomy was slightly lower in the 1958 cohort than in the 1946 cohort (Table 2). Women in the 1958 cohort had higher mean BMI, mean waist circumference, educational levels and prevalence of nulliparity than women in the 1946 cohort.

In unadjusted analyses using the maximum available samples, we found that women who had previously undergone hysterectomy with or without oophorectomy had higher BMI and waist circumference on average than women who had not undergone hysterectomy or oophorectomy. The differences in mean BMI were 1.18 kg/m^2^ (95% CI: 0.64, 1.74) at age 44–45 years in the 1958 cohort (*N* = 4477, number of hysterectomies = 445); 0.76 kg/m^2^ (−0.05, 1.57) at age 43 years (*N* = 1582, number of hysterectomies = 145) and 0.81 kg/m^2^ (0.14, 1.49) at age 53 years (*N* = 1466, number of hysterectomies = 314) in the 1946 cohort. The differences in mean waist circumference were 2.72 cm (1.45, 3.99) at age 44–45 years in the 1958 cohort (*N* = 4449, number of hysterectomies = 441); 0.34 cm (−1.58, 2.26) at age 43 years (*N* = 1581, number of hysterectomies = 144) and 1.45 cm (−0.15, 3.05) at age 53 years (*N* = 1476, number of hysterectomies = 315) in the 1946 cohort.

In both cohorts results of likelihood ratio tests suggested that categorising hysterectomy by any one of the characteristics did not significantly improve model fit. There was however some evidence that women who had undergone hysterectomy before age 40 years had a slightly higher subsequent BMI and waist circumference than women who had not had hysterectomy or oophorectomy (Table 3 shows results for BMI). In the 1946 cohort, we also found a suggestion that women who had undergone hysterectomy for fibroids or had undergone a vaginal hysterectomy had higher subsequent adiposity than other women.

After individual adjustments for ‘pre-hysterectomy’ BMI, age at menarche, parity and lifetime socioeconomic position, differences in mean BMI between the women who had undergone hysterectomy and those who had not attenuated (Table 4). For example, adjustment for BMI at age 26 years reduced the difference in mean BMI at age 53 years by 44% in the 1946 cohort and adjustment for BMI at age 23 years reduced the difference in mean BMI at age 44–45 years by 37% in the 1958 cohort. Adjustment for age at menarche reduced the difference in mean BMI at age 53 years by 26% in the 1946 cohort and the difference in mean BMI at age 44–45 years by 13% in the 1958 cohort. Conversely, in the 1946 cohort adjustment for HRT use at both ages of outcome and for exercise levels where 43 years was the age of outcome amplified the differences in mean BMI. There was no evidence of interactions between hysterectomy and pre-hysterectomy BMI or HRT use. In multivariable models, after adjusting for measures of ‘pre-hysterectomy’ BMI there were only small effects of adjusting for additional covariates. In models adjusting for all covariates simultaneously there were no significant differences in mean BMI, differences in mean BMI at age 53 years in the 1946 cohort were 0.43 kg/m^2^ (−0.25, 1.11) (*N* = 963, number of hysterectomies = 207) and at age 44–45 years in the 1958 cohort were 0.42 kg/m^2^ (−0.22, 1.07) (*N* = 2021, number of hysterectomies = 192).

Differences in mean waist circumference between the women who had undergone hysterectomy and those who had not also attenuated after individual adjustments for all confounders in the 1958 cohort and for most confounders in the 1946 cohort (Table 5). For example, adjustment for age at menarche resulted in a 25% reduction in the difference in mean waist circumference at age 53 years in the 1946 cohort and a 10% reduction at age 44–45 years in the 1958 cohort. Adjustment for lifetime socioeconomic position reduced differences in mean waist circumference by 18% at age 53 years in the 1946 cohort and by 25% in the 1958 cohort. Exceptions in the 1946 cohort were the adjustments for HRT use at both ages of outcome, and exercise levels, where 43 years was the age of outcome, both of which amplified differences in mean waist circumference. As for BMI, there was no evidence of interactions and in models adjusting for all covariates simultaneously there were no significant differences in mean waist circumference by hysterectomy status in either cohort. In a sub-group analyses in the 1946 cohort (*N* = 1249), we examined the association between hysterectomy and waist circumference at age 53 years adjusted for waist circumference at age 43 years, excluding women whose hysterectomies were before age 43 years: the mean difference in waist circumference at age 53 years between the women who had and had not undergone hysterectomy decreased from 1.00 cm (−1.14, 3.15) to 0.35 cm (−0.98, 1.68).

## 4 Discussion

### 4.1 Main findings

In two large British birth cohorts, results from unadjusted analyses showed that women who had previously undergone hysterectomy had higher BMI and waist circumference in middle-age than women who had not undergone hysterectomy or oophorectomy. However, these differences in adiposity were modest and appeared to be explained by pre-hysterectomy BMI and the greater exposure of women who had undergone hysterectomy to risk factors for increased adiposity. Results were consistent across birth cohorts despite differences in the prevalence of hysterectomy, mean BMI and waist circumference and other characteristics between cohorts.

### 4.2 Comparison with other studies

Few previous studies had designs that were appropriate to test whether hysterectomy predicts subsequent adiposity, with different studies producing results that were not fully consistent with each other. This study, especially the prospective examination of BMI, is an improvement on previous research, most of which is cross-sectional. While associations between hysterectomy and subsequent BMI and waist circumference in unadjusted analyses were observed in both cohorts, supporting the findings from some previous studies [1,2,13] our analyses suggest that the association was largely due to differences in pre-hysterectomy adiposity and confounding factors, which many other studies had not considered.

The attenuation in the size of the associations between hysterectomy and subsequent BMI and waist circumference after control for pre-hysterectomy BMI and confounders supports other recent research which suggests that women who undergo hysterectomy have higher levels of risk factors for poor health than other women and that this may be responsible for associations between hysterectomy and subsequent health rather than because there is a direct detrimental effect of hysterectomy [4,18,19]. It is also consistent with our recent findings suggesting that women in the 1946 cohort who underwent hysterectomy may already have been on a trajectory of increased weight gain prior to surgery [8].

### 4.3 Methodological considerations

There are several important strengths of this study. One of these is the temporal nature of the data, which enables an examination of the association between hysterectomy and subsequent BMI taking into account BMI prior to hysterectomy. Another is that in both cohorts, the outcome measures and one pre-hysterectomy measure of waist circumference, height and weight were recorded by trained professionals thus avoiding bias which various studies have shown is introduced if self-reported measures of these variables are used [20–23]. The wealth of information about both cohorts made it possible to adjust for a range of factors collected prospectively across life greatly reducing the impact of recall bias and the potential for residual confounding.

A further strength is that within each of the two cohorts participants were the same age. Hence, factors that could influence the associations under investigation, such as social and cultural changes over time (e.g. changes in women's social roles, educational levels, childbearing, medical practices and oral contraceptive and HRT use) and period effects (e.g. the rise in overweight and obesity) were constant within each cohort. Using data from two cohorts is strength of this study as it demonstrates that the results are generalisable to women of different ages who have experienced different rates of hysterectomy, levels of overweight and obesity, parity and education (clearly shown in Table 2). The results are also likely to be generalisable as both cohorts were selected to be nationally representative and remain so in most respects despite losses to follow-up [24,25].

There are also several limitations to this study. To assess the likely effects of hysterectomy on health across the remainder of life, assessing the effect of hysterectomy on measures of central adiposity may be more relevant than assessing the effect of hysterectomy on BMI; recent studies have shown that waist circumference is a better predictor of subsequent chronic disease risk than BMI [26,27]. Pre-hysterectomy measures of waist circumference were not available in the 1958 cohort and were only available at age 43 years in the 1946 cohort and so the main focus of analyses was on BMI. Although BMI does not provide an indication of body fat distribution or distinguish between fat and lean mass, it is regarded as an appropriate measure for capturing most of the relevant variation in overall levels of adiposity [15,28,29] and furthermore, results from comparable analyses of BMI and waist circumference were similar.

While it was possible to adjust for ‘pre-hysterectomy’ BMI in analyses of the association between hysterectomy and subsequent BMI none of the measures used were ideal. The measures of BMI at 26 and 23 years, did precede hysterectomy: in the 1946 cohort all hysterectomies occurred after age 26 years and in the 1958 cohort only two hysterectomies occurred at or before age 23 years. However, these ages were well in advance of the majority of hysterectomies and hence there may have been an under-adjustment of BMI as it increased across adulthood (Table 2). For this reason adjustments were also made for BMI at a later age, i.e. at ages 36 and 33 years in the 1946 and 1958 cohorts, respectively. Although the majority of hysterectomies (86% in the 1946 cohort and 80% in the 1958 cohort) occurred after these measures were taken, for some women this was a post-hysterectomy measure of BMI and so may represent an over-adjustment. The true effect of adjusting for ‘pre-hysterectomy’ BMI therefore probably lies somewhere between the two.

By excluding women who had been lost to follow-up and who had missing data on variables of interest, bias could have been introduced. In the 1946 cohort, women lost to follow-up had lower educational levels and higher age at menarche than women who were included but there were no significant differences in BMI, occupational class or parity. In the 1958 cohort, women lost to follow-up had lower socioeconomic position, higher BMI and later age at menarche than women included in the study sample. These differences were small and so the bias introduced by sample attrition is not expected to be large. As well as potentially introducing bias, sample attrition and exclusions have reduced the size of the samples available for analysis. This could have resulted in there being insufficient power to detect the levels of effect, which existed; this is especially true in analyses of variation in outcome by characteristics of hysterectomy.

## 5 Conclusions

This study demonstrates that British women who had previously undergone hysterectomy had higher BMI and waist circumference in middle age than others. These differences appear to be accounted for by the higher BMI in earlier adulthood and increased levels of risk factors associated with both adiposity and hysterectomy risk among women who had undergone hysterectomy. Women can thus be reassured that they are unlikely to experience weight gain as a direct result of hysterectomy. Women who have undergone hysterectomy do however represent a defined group who could be targeted with advice about weight control.

## Conflict of interest

None.

## Ethical approval

Ethical approval for the most recent data collection, at age 53 years, of the 1946 cohort was issued by North Thames Multi-centre Research Ethics Committee, and cohort members gave informed consent for every aspect of the data collection procedure. Ethical approval for the biomedical study of the 1958 birth cohort was obtained from the South East Multi-centre Research Ethics Committee.

## Figures and Tables

**Table 1:** Summary of ages and methods of collection of data used in analyses

	1946 cohort		1958 cohort	
	Age/s (years) at collection	Method of collection	Age(s) (years) at collection	Method of collection
Outcome measures				
Body mass index (kg/m^2^)	43 and 53	Height and weight measured by nurses during home visit	44–45	Height and weight measured by nurses during home visit
Waist circumference (cm)	43 and 53	Measured by nurses during home visit	44–45	Measured by nurses during home visit

Main explanatory variable				
Hysterectomy status	43 and 53	Home visit questionnaire	44–45	Home visit questionnaire
	47 to 54 and 57	Annual postal questionnaires		

Covariates				
Pre-hysterectomy body mass index	26 and 36	Height and weight self-reported at age 26, measured by nurses at age 36	23 and 33	Height and weight self-reported at age 23, measured by interviewers at age 33
Parity	Up to 53	Self-reported in questionnaires	Up to 44–45	Self-reported in questionnaires
Age at menarche	14–15	Medical interview	16	Parental questionnaires
Father's occupational class	11	Parental questionnaires	11	Parental questionnaires
Own occupational class	43	Self-reported in questionnaire	42	Self-reported in questionnaire
Educational level	26	Self-reported in questionnaire	33	Self-reported in questionnaire
Exercise levels	36, 43 and 53	Self-reported in questionnaire [30]	33 and 42	Self-reported in questionnaire [31]
Hormone replacement therapy use	Up to 53	Self-reported in questionnaire	44–45	Self-reported in questionnaire

**Table 2:** Characteristics of the study populations

	Mean (S.D.) [*N*] or *n* (%)	
	1946 cohort	1958 cohort
Total *N* with data on hysterectomy	1790	4552

Prevalence of hysterectomy up to and including age (years)^a^		
30	14 (0.8)	48 (1.1)
35	47 (2.6)	147 (3.2)
40	126 (7.0)	298 (6.5)
44	209 (11.7)	437 (9.6)
50	337 (18.8)	–
55	393 (22.0)	–

Body mass index (kg/m^2^) at age (years)		
15	20.7 (3.0) [1336]	–
16	–	21.0 (2.9) [3327]
20	21.9 (2.9) [1427]	–
23	–	22.0 (3.1) [3933]
26	22.4 (3.3) [1534]	–
33	–	24.4 (4.6) [4067]
36	23.6 (4.0) [1560]	–
43	25.0 (4.8) [1608]	–
44/45	–	27.0 (5.6) [4544]
53	27.5 (5.5) [1489]	–

Waist circumference (cm) at age (years)		
43	77.9 (11.3) [1608]	–
44/45	–	85.5 (12.9) [4515]
53	85.9 (12.9) [1499]	–

Father's occupational class in childhood^b^		
I or II	431 (25.5)	1085 (25.1)
IIINM	276 (16.3)	424 (9.8)
IIIM	546 (32.3)	1790 (41.4)
IV or V	436 (25.8)	1026 (23.7)

Educational level attained		
University degree or higher	91 (5.4)	1141 (28.1)
Advanced secondary qualifications^c^	374 (22.3)	439 (10.8)
Ordinary secondary qualifications^d^	420 (25.0)	1510 (37.2)
Below secondary qualifications	157 (9.4)	646 (15.9)
None	637 (37.9)	325 (8.0)

Own occupational class in adulthood^b^		
I or II	588 (35.8)	1613 (37.2)
IIINM	611 (37.2)	1468 (33.9)
IIIM	120 (7.3)	322 (7.4)
IV or V	323 (19.7)	930 (21.5)

Age at menarche (years)		
≤11	235 (16.4)	458 (15.7)
12	397 (27.7)	712 (24.4)
13	487 (34.0)	1018 (34.8)
≥14	315 (22.0)	735 (25.2)

Parity (at age 43 years in 1946 cohort and 42 years in 1958 cohort)		
0	197 (12.2)	713 (17.7)
1	209 (12.9)	650 (16.2)
2	714 (44.0)	1718 (42.7)
3	353 (21.8)	722 (18.0)
≥4	149 (9.2)	220 (5.5)

Exercise (at age 36 years in 1946 cohort and 33 years in 1958 cohort)		
	None 661 (42.0)	≤2–3 times per month 1243 (30.2)
	1–4 times per month 380 (24.1)	once a week 939 (22.8)
	>4 times per month 533 (33.9)	Two to three times per week 846 (20.5)
		4–7 times per week 1093 (26.5)

HRT use (at age 53 years in 1946 cohort and 44–45 years in 1958 cohort)		
Never	652 (43.1)	3955 (88.6)
Ex-user	303 (20.0)	178 (4.0)
Current user	557 (36.8)	331 (7.4)

a *N* and % are cumulative.

b Registrar general's social classification: I or II professional, managerial or technical; IIINM non-manual; IIIM manual skilled; IV or V partly skilled or unskilled.

c Generally taken at age 18 years.

d Generally taken at age 16 years.

**Table 3:** Regression coefficients and 95% confidence intervals for the association between characteristics of hysterectomy and BMI (kg/m^2^) at ages 43 and 53 years in the 1946 cohort and at age 44/45 years in the 1958 cohort

	1946 cohort				1958 cohort	
	*N*	Regression coefficient (difference in mean BMI at age 43 years) (95% CI)	*N*	Regression coefficient (difference in mean BMI at age 53 years) (95% CI)	*N*	Regression coefficient (difference in mean BMI at age 44/45 years) (95% CI)
No hysterectomy or oophorectomy	1446	Reference group	1154	Reference group	4032	Reference group
Hysterectomy with oophorectomy^a^	42	0.82 (−0.64, 2.29)	166	0.73 (−0.15, 1.62)	228	1.40 (0.65, 2.15)
Hysterectomy no oophorectomy	103	0.70 (−0.26, 1.65)	148	0.89 (−0.05, 1.82)	217	0.96 (0.20, 1.73)
Oophorectomy only^a^	16	−1.11 (−3.46, 1.24)	20	0.77 (−1.65, 3.18)	63	−0.82 (−2.22, 0.58)
*p*-Value*		0.88		0.80		0.41

No hysterectomy or oophorectomy	1437	Reference group	1152	Reference group	4032	Reference group
Age at hysterectomy (years)						
<40	95	1.15 (0.16, 2.13)	87	1.12 (−0.06, 2.31)	255	1.37 (0.66, 2.08)
40–44	50	0.02 (−1.31, 1.36)	81	0.89 (−0.34, 2.11)	190	0.94 (0.13, 1.76)
45–49	–	–	100	0.55 (−0.56, 1.66)	–	–
≥50	–	–	46	0.68 (–0.93, 2.28)	–	–
*p*-Value*		0.17		0.90		0.43

No hysterectomy or oophorectomy	1437	Reference group	1152	Reference group		–
Hysterectomy for						
Fibroids	36	1.28 (−0.29, 2.84)	105	1.25 (0.16, 2.34)		
Menstrual disorders	51	0.29 (−1.04, 1.61)	98	0.52 (−0.60, 1.64)		
Prolapse	7	0.59 (−2.93, 4.11)	28	−0.67 (−2.71, 1.37)		
Cancer	15	−0.23 (−2.64, 2.18)	19	0.62 (−1.85, 3.08)		
Other reasons	29	1.63 (−0.12, 3.37)	48	1.17 (−0.40, 2.74)		
Unknown reasons	7	0.25 (−3.28, 3.77)	16	1.54 (−1.14, 4.23)		
*p*-Value*		0.75		0.61		

No hysterectomy or oophorectomy	1437	Reference group	1152	Reference group		–
Route of hysterectomy						
Abdominal	106	0.59 (−0.34, 1.53)	239	0.60 (−0.16, 1.35)		
Vaginal	19	2.70 (0.55, 4.84)	56	1.51 (0.05, 2.97)		
Unknown	20	−0.19 (−2.28, 1.90)	19	1.48 (−0.98, 3.94)		
*p*-Value*		0.13		0.45		

Regression coefficients represent the differences in mean BMI (kg/m^2^) between the women who had undergone hysterectomy by the time of BMI outcome measurement, categorised by characteristics of hysterectomy, and women who had not undergone hysterectomy or oophorectomy by the time of BMI outcome measurement.

a Bilateral or unilateral oophorectomy.

* *p*-Values from likelihood ratio tests comparing model with categorisation of hysterectomy shown with a model in which all hysterectomies were grouped together (women with oophorectomy only excluded from all models except those examining oophorectomy status).

**Table 4:** Regression coefficients and 95% confidence intervals for the association between hysterectomy and BMI (kg/m^2^) at ages 43 and 53 years in the 1946 cohort and at age 44/45 years in the 1958 cohort adjusted separately for: (a) pre-hysterectomy BMI, (b) age at menarche, (c) parity, (d) lifetime socioeconomic position, (e) exercise in adulthood and (f) hormone replacement therapy use

Adjusted for	1946 cohort				1958 cohort	
	Regression coefficient (difference in mean BMI at age 43 years) (95% CI)	*p*-Value*	Regression coefficient (difference in mean BMI at age 53 years) (95% CI)	*p*-Value*	Regression coefficient (difference in mean BMI at age 44/45 years) (95% CI)	*p*-Value*
BMI at age 26 or 23 years	*N* = 1378		*N* = 1277		*N* = 3864	
Unadjusted	0.97 (0.10, 1.85)	0.03	0.79 (0.07, 1.52)	0.03	1.04 (0.46, 1.63)	0.001
Adjusted	0.88 (0.27, 1.49)	0.01	0.44 (−0.12, 1.01)	0.13	0.66 (0.24, 1.08)	0.002

BMI at age 36 or 33 years	*N* = 1444		*N* = 1325		*N* = 3998	
Unadjusted	0.83 (−0.001, 1.66)	0.05	0.91 (0.21, 1.61)	0.01	1.16 (0.58, 1.74)	<0.001
Adjusted	0.52 (0.11, 0.94)	0.01	0.34 (−0.10, 0.78)	0.13	0.82 (0.47, 1.17)	<0.001

Age at menarche	*N* = 1276		*N* = 1196		*N* = 2872	
Unadjusted	0.48 (−0.43, 1.39)	0.30	0.70 (−0.07, 1.46)	0.07	1.14 (0.48, 1.81)	0.001
Adjusted	0.22 (−0.67, 1.10)	0.63	0.52 (−0.23, 1.27)	0.18	0.99 (0.34, 1.63)	0.003

Parity	*N* = 1581		*N* = 1465		*N* = 3951	
Unadjusted	0.76 (−0.05, 1.56)	0.07	0.81 (0.13, 1.49)	0.02	1.17 (0.58, 1.77)	<0.001
Adjusted	0.74 (−0.07, 1.55)	0.07	0.74 (0.05, 1.42)	0.03	1.16 (0.57, 1.75)	<0.001

Lifetime socioeconomic position	*N* = 1368		*N* = 1277		*N* = 3700	
Unadjusted	0.74 (−0.09, 1.57)	0.08	0.97 (0.24, 1.69)	0.01	0.91 (0.30, 1.52)	0.004
Adjusted	0.60 (−0.22, 1.42)	0.15	0.82 (0.10, 1.53)	0.03	0.70 (0.09, 1.31)	0.03

Exercise in adulthood	*N* = 1456		*N* = 1337		*N* = 3950	
Unadjusted	0.81 (−0.03, 1.65)	0.06	0.86 (0.15, 1.56)	0.02	1.21 (0.61, 1.80)	<0.001
Adjusted	0.87 (0.04, 1.69)	0.04	0.81 (0.12, 1.49)	0.02	1.11 (0.52, 1.70)	<0.001

Hormone replacement therapy use	*N* = 1404		*N* = 1466		*N* = 4452	
Unadjusted	1.01 (0.14, 1.88)	0.02	0.81 (0.14, 1.49)	0.02	1.15 (0.59, 1.70)	<0.001
Adjusted	1.24 (0.36, 2.12)	0.01	1.25 (0.54, 1.96)	0.001	0.85 (0.25, 1.46)	0.006

Regression coefficients represent the differences in mean BMI (kg/m^2^) between the women who had undergone hysterectomy by the time of BMI outcome measurement and women who had not undergone hysterectomy or oophorectomy by the time of BMI outcome measurement. *Note 1*: The reference group is women who had not undergone hysterectomy or oophorectomy by the time of BMI outcome measurement. *Note 2*: The same *N* is used for unadjusted analysis as is used for the corresponding adjusted analysis to ensure comparability of models

* *p*-Values from significance tests of the differences in mean BMI.

**Table 5:** Regression coefficients and 95% confidence intervals for the association between hysterectomy and waist circumference (WC) (cm) at ages 43 and 53 years in the 1946 cohort and at age 44/45 years in the 1958 cohort adjusted separately for: (a) age at menarche, (b) parity, (c) lifetime socioeconomic position, (d) exercise in adulthood and (e) hormone replacement therapy use

Adjusted for	1946 cohort				1958 cohort	
	Regression coefficient (difference in mean WC at age 43 years) (95% CI)	*p*-Value*	Regression coefficient (difference in mean WC at age 53 years) (95% CI)	*p*-Value*	Regression coefficient (difference in mean WC at age 44/45y) (95% CI)	*p*-Value*
Age at menarche	*N* = 1275		*N* = 1202		*N* = 2854	
Unadjusted	0.27 (−1.87, 2.41)	0.81	1.06 (−0.72, 2.85)	0.24	2.57 (1.05, 4.09)	0.001
Adjusted	−0.14 (−2.26, 1.98)	0.90	0.79 (−0.99, 2.57)	0.38	2.31 (0.82, 3.81)	0.002

Parity	*N* = 1580		*N* = 1475		*N* = 3925	
Unadjusted	0.33 (−1.59, 2.25)	0.74	1.44 (−0.16, 3.04)	0.08	2.64 (1.28, 4.00)	<0.001
Adjusted	0.27 (−1.65, 2.19)	0.78	1.24 (−0.37, 2.84)	0.13	2.61 (1.25, 3.97)	<0.001

Lifetime socioeconomic position	*N* = 1366		*N* = 1285		*N* = 3682	
Unadjusted	0.44 (−1.55, 2.44)	0.67	1.85 (0.13, 3.57)	0.04	2.04 (0.63, 3.46)	0.005
Adjusted	0.06 (−1.91, 2.03)	0.95	1.52 (−0.17, 3.21)	0.08	1.54 (0.13, 2.95)	0.03

Exercise in adulthood	*N* = 1455		*N* = 1347		*N* = 3926	
Unadjusted	0.66 (−1.35, 2.67)	0.52	1.66 (−0.004, 3.33)	0.05	2.51 (1.14, 3.88)	<0.001
Adjusted	0.83 (−1.13, 2.80)	0.41	1.53 (−0.09, 3.15)	0.06	2.30 (0.94, 3.66)	0.001

Hormone replacement therapy use	*N* = 1402		*N* = 1476		*N* = 4424	
Unadjusted	0.70 (−1.38, 2.77)	0.51	1.45 (−0.15, 3.05)	0.08	2.61 (1.33, 3.89)	<0.001
Adjusted	1.08 (−1.02, 3.18)	0.31	2.32 (0.64, 4.00)	0.01	1.99 (0.58, 3.39)	0.005

Regression coefficients represent the differences in mean WC (cm) between the women who had undergone hysterectomy by the time of WC outcome measurement and women who had not undergone hysterectomy or oophorectomy by the time of WC outcome measurement. *Note 1*: The reference group is women who had not undergone hysterectomy or oophorectomy by the time of WC outcome measurement. *Note 2*: The same *N* is used for unadjusted analysis as is used for the corresponding adjusted analysis to ensure comparability of models.

* *p*-Values from significance tests of the differences in mean waist circumference.

## References

[1] Matthews K.A., Abrams B., Crawford S. (2001). Body mass index in mid-life women: relative influence of menopause, hormone use, and ethnicity. Int J Obes.

[2] Lambert L.J., Straton J.A.Y., Knuiman M.W., Bartholomew H.C. (2003). Health status of users of hormone replacement therapy by hysterectomy status in Western Australia. J Epidemiol Community Health.

[3] Farquhar C.M., Sadler L., Harvey S.A., Stewart A.W. (2005). The association of hysterectomy and menopause: a prospective cohort study. BJOG.

[4] Howard B.V., Kuller L., Langer R. (2005). Risk of cardiovascular disease by hysterectomy status, with and without oophorectomy: the women's health initiative observational study. Circulation.

[5] Lake J.K., Power C., Cole T.J. (1997). Women's reproductive health: the role of body mass index in early and adult life. Int J Obes.

[6] Xu W.H., Matthews C.E., Xiang Y.B. (2005). Effect of adiposity and fat distribution on endometrial cancer risk in Shanghai women. Am J Epidemiol.

[7] Settnes A., Jorgensen T., Lange A.P. (1996). Hysterectomy in Danish women: weight-related factors, psychologic factors, and life-style variables. Obstet Gynecol.

[8] Cooper R, Hardy R, Kuh D. Is adiposity across life associated with subsequent hysterectomy risk? Findings from the 1946 British birth cohort study. BJOG; in press. 10.1111/j.1471-0528.2007.01569.x 18081600

[9] Hjortland M.C., McNamara P.M., Kannel W.B. (1976). Some atherogenic concomitants of menopause: the Framingham study. Am J Epidemiol.

[10] Akahoshi M., Soda M., Nakashima E., Shimaoka K., Shinji S., Katsusuke Y. (1996). Effects of menopause on trends of serum cholesterol, blood pressure, and body mass index. Circulation.

[11] Sowers M.F., Crutchfield M., Jannausch M.L., Russell-Aulet M. (1996). Longitudinal changes in body composition in women approaching the midlife. Ann Hum Biol.

[12] Stoney C.M., Owens J.F., Guzick D.S., Matthews K.A. (1997). A natural experiment on the effects of ovarian hormones on cardiovascular risk factors and stress reactivity: bilateral salpingo oophorectomy versus hysterectomy only. Health Psychol.

[13] Kirchengast S., Gruber D., Sator M., Huber J. (2000). Hysterectomy is associated with postmenopausal body composition characteristics. J Biosoc Sci.

[14] Redburn J.C., Murphy M.F.G. (2001). Hysterectomy prevalence and adjusted cervical and uterine cancer rates in England and Wales. Br J Obstet Gynaecol.

[15] Power C., Parsons T., Kuh D., Hardy R. (2002). Overweight and obesity from a life course perspective. A life course approach to women's health.

[16] Wadsworth M., Kuh D., Richards M., Hardy R. (2006). Cohort profile: the 1946 National birth cohort (MRC National Survey of Health and Development). Int J Epidemiol.

[17] Power C., Elliott J. (2006). Cohort profile: 1958 British birth cohort (National Child Development Study). Int J Epidemiol.

[18] Brett K.M. (2005). Can hysterectomy be considered a risk factor for cardiovascular disease?. Circulation.

[19] Ceausu I., Shakir Y.A., Lidfeldt J., Samsioe G., Nerbrand C. (2006). The hysterectomized woman—is she special? The women's health in the Lund area (WHILA) study. Maturitas.

[20] Niedhammer I., Bugel I., Bonenfant S., Goldberg M., Leclerc A. (2000). Validity of self-reported weight and height in the French GAZEL cohort. Int J Obes.

[21] Spencer E.A., Appleby P.N., Davey G.K., Key T.J. (2002). Validity of self-reported height and weight in 4808 EPIC-Oxford participants. Public Health Nutr.

[22] Elgar F.J., Roberts C., Tudor-Smith C., Moore L. (2005). Validity of self-reported height and weight and predictors of bias in adolescents. J Adolesc Health.

[23] Bigaard J., Spanggaard I., Thomsen B.L., Overvad K., Tjonneland A. (2005). Self-reported and technician-measured waist circumferences differ in middle-aged men and women. J Nutr.

[24] Wadsworth M.E.J., Butterworth S.L., Hardy R.J. (2003). The life course prospective design: an example of benefits and problems associated with longevity. Soc Sci Med.

[25] Atherton K, Fuller E, Shepherd P, Strachan D, Power C. Loss and representativeness in a biomedical survey at age 45 years: 1958 British birth cohort. J Epidemiol Community Health; in press. 10.1136/jech.2006.058966 18272736

[26] Kragelund C., Omland T. (2005). A farewell to body-mass-index?. Lancet.

[27] Yusuf S., Hawken S., Ounpuu S. (2005). Obesity and the risk of myocardial infarction in 27,000 participants from 52 countries: a case–control study. Lancet.

[28] Gillman M.W., Kuh D., Ben-Shlomo Y. (2004). A life course approach to obesity. A life course approach to chronic disease epidemiology.

[29] Snijder M.B., van Dam R.M., Visser M., Seidell J.C. (2006). What aspects of body fat are particularly hazardous and how do we measure them?. Int J Epidemiol.

[30] Kuh D.J.L., Cooper C. (1992). Physical activity at 36 years: patterns and childhood predictors in a longitudinal study. J Epidemiol Community Health.

[31] Parsons T.J., Manor O., Power C. (2005). Changes in diet and physical activity in the 1990s in a large British sample (1958 birth cohort). Eur J Clin Nutr.

